# The Therapeutic Potential of GLP-1 Receptor Agonists in the Management of Hidradenitis Suppurativa: A Systematic Review of Anti-Inflammatory and Metabolic Effects

**DOI:** 10.3390/jcm13216292

**Published:** 2024-10-22

**Authors:** Piotr K. Krajewski, Aleksandra Złotowska, Jacek C. Szepietowski

**Affiliations:** 1 University Centre of General Dermatology and Oncodermatology, Wroclaw Medical University, 50-556 Wroclaw, Poland; piotr.krajewski@umw.edu.pl (P.K.K.); aleksandra.zlotowska@student.umw.edu.pl (A.Z.); 2 Faculty of Medicine, Wroclaw University of Science and Technology, 51-377 Wroclaw, Poland

**Keywords:** GLP-1, GLP-1 agonists, hidradenitis suppurativa, acne inversa

## Abstract

**Background:** Glucagon-like peptide-1 receptor agonists (GLP1-RAs) are synthetic peptides that mimic the natural activity of GLP-1, widely known for lowering blood glucose levels and promoting weight reduction. These characteristics make them a valuable tool in managing type 2 diabetes and obesity-related conditions. Recent findings indicate that GLP1-RAs may also offer therapeutic benefits in managing hidradenitis suppurativa (HS), a chronic inflammatory skin disorder closely associated with metabolic abnormalities, including obesity, diabetes, and dyslipidemia. This review explores the potential role of GLP1-RAs in managing HS. **Methods:** A systematic review was conducted by searching electronic databases, including MEDLINE and Google Scholar, without date limitations. Key search terms included “GLP-1” or “GLP-1 agonists” combined with “hidradenitis suppurativa” or “acne inversa”. Inclusion criteria were set for studies reporting on the use of GLP1-RAs as a treatment for HS, with articles discussing theoretical applications excluded. Data synthesis included findings from 25 relevant studies. **Results:** The analysis revealed that GLP1-RAs, specifically liraglutide and semaglutide, led to significant reductions in weight and systemic inflammation in HS patients. Notably, improvements in lesion severity and quality of life were reported. The anti-inflammatory effects of GLP1-RAs were attributed to the suppression of key inflammatory pathways involving TNF-α, IL-17, and NF-κB. **Conclusions:** GLP1-RAs demonstrate significant potential as an adjunct therapy for HS, addressing both the metabolic and inflammatory aspects of the condition. While early results are promising, further research is necessary to determine their long-term efficacy in managing HS.

## 1. Introduction

Glucagon-like peptide-1 (GLP-1) receptor agonists (GLP1-RAs), also known as GLP-1 analogs, are synthetically produced recombinant polypeptides that mimic the activity of GLP-1. These agents are derived from either exendin or human GLP-1 and are designed to have enhanced enzymatic stability against dipeptidyl peptidase 4 (DPP-4), along with slower elimination kinetics compared to native GLP-1. As a result, GLP1-RAs have a prolonged duration of action [1,2]. These drugs effectively lower glucose levels and reduce body weight, making them valuable in treating type II diabetes, obesity, and dyslipidemia, as well as other metabolic diseases associated with obesity. Additionally, GLP1-RAs exhibit anti-inflammatory properties that are independent of glycemic control [3].

Hidradenitis suppurativa (HS) is a chronic inflammatory skin condition characterized by painful, deep-seated, purulent lesions primarily found in intertriginous areas [4]. Patients with HS are at an increased risk of developing obesity, dyslipidemia, and type II diabetes compared to the general population. These metabolic abnormalities contribute to the increased inflammation that plays a key role in the pathogenesis of the disease [5]. Metainflammation is chronic low-grade systemic inflammation driven by metabolic dysregulation, often seen in conditions like obesity, diabetes, and HS. This inflammation is closely linked to adipose tissue dysfunction and systemic insulin resistance, contributing to the exacerbation of HS symptoms [5,6]. Emerging evidence suggests that HS itself is an independent risk factor for obesity, as seen in metabolically unhealthy lean (MUL) individuals with HS [6]. These lean HS patients often exhibit markers of metabolic syndrome, such as insulin resistance, dyslipidemia, and elevated inflammatory markers, despite having a normal BMI. This challenges the conventional view that obesity alone drives metabolic complications in HS, underscoring the metabolic burden HS imposes independently of body weight [6].

HS remains a challenging condition to treat due to its chronic, relapsing nature. Current treatments include a range of medical and surgical approaches, such as topical and systemic antibiotics, retinoids, hormonal therapies, and biologics like TNF and IL-17 inhibitors (e.g., adalimumab, secukinumab, bimekizumab) [6]. Despite the availability of these treatments, many patients experience suboptimal outcomes due to a combination of factors. Antibiotic resistance, incomplete disease remission, and frequent recurrences are commonly reported, limiting the long-term efficacy of these therapies [6]. Moreover, while effective for some, biologics often fail to achieve sustained disease control and are associated with significant costs, potential side effects, and limited accessibility in some regions [6]. The primary reason these treatments are often inadequate is the heterogeneous nature of the disease, which involves multiple pathogenic pathways, including follicular occlusion, immune dysregulation, and genetic predisposition [6,7,8]. As a result, many patients do not fully respond to available therapies, highlighting the urgent need for more targeted, personalized treatment strategies that address the multifactorial pathogenesis of HS. Current research into new treatments for HS, focused on biological drugs and small molecules, aims to target specific inflammatory cytokines to disturb the inflammatory loop. Nevertheless, researchers indicated that regardless of the treatment, lifestyle changes, including tobacco cessation and loss of body weight, could significantly decrease the disease severity and prolong the periods between flare ups [7,8]. Moreover, recognizing metainflammation as a therapeutic target is crucial, as it opens up new avenues for treatment strategies, such as using GLP-1 receptor agonists, which have both anti-inflammatory and weight-reducing properties that directly address these underlying metabolic abnormalities. Introducing this concept will provide a more comprehensive framework for discussing the potential benefits of targeting metabolic pathways in HS management.

Recent advancements in the use of GLP-1 agonists, particularly for weight management, have highlighted their potential as a game-changer in treating obesity and related metabolic conditions [9]. GLP-1 agonists could help manage chronic inflammatory skin disorders by reducing systemic inflammation and improving insulin sensitivity. While still in the early stages of investigation, these advancements point to a promising new application of GLP-1 agonists in dermatology, potentially offering a novel approach to managing chronic skin disorders [10].

With this in mind, we decided to perform a systematic review exploring the potential of GLP-1 analogs in treating HS, highlighting their promising therapeutic benefits due to their anti-inflammatory effects and proven efficacy in weight reduction.

## 2. Methodology and Search Strategy

**Figure 1:**
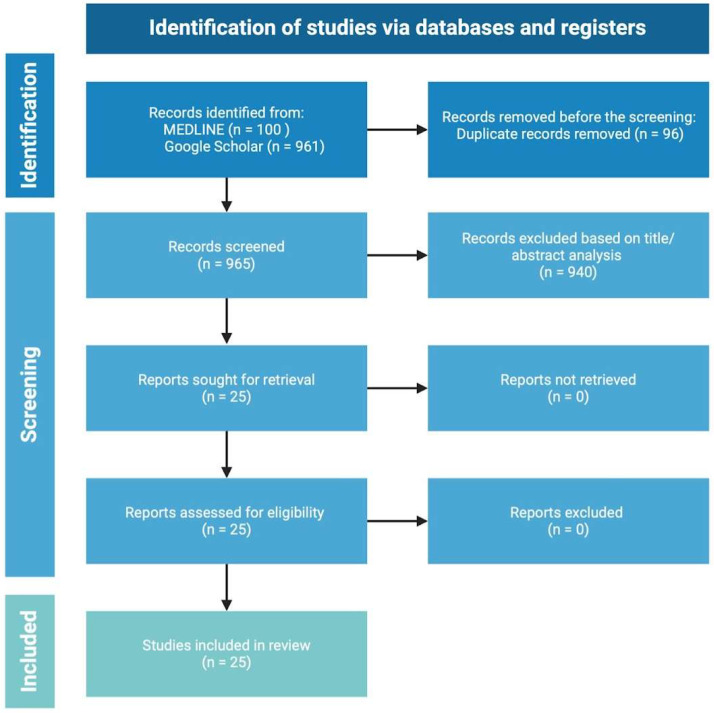
Literature search carried out according to the PRISMA guidelines.

Two electronic databases (MEDLINE and Google Scholar) were systematically searched without setting data limitations in June 2024 according to the PRISMA guidelines [11]. The main keywords used were “GLP-1” or “GLP-1 agonists” AND “hidradenitis suppurativa” or “acne inversa” or “HS”. The studies were not restricted by language and were limited to human subjects. The search was performed by two independent authors (PKK and AZ). The inclusion criteria were that all studies collected empirical data on the use of GLP-1 agonists as a main or supportive treatment for HS. Articles regarding the rationale behind the use of GLP-1 agonists to treat HS were excluded. The absence of clinical trials and comparative studies evaluating the efficacy of GLP-1 agonists in treating HS has hindered the ability to conduct a meta-analysis. In the final review, 25 articles were included. The graphical presentation of the search strategy is presented in Figure 1.

## 3. Hidradenitis Suppurativa

Hidradenitis suppurativa (HS) is a chronic and debilitating inflammatory skin disorder that affects approximately 0.5–1% of the population [12]. Clinically, HS is characterized by painful, deep-seated abscesses primarily located in intertriginous areas [13]. HS is marked by severe pain and persistent purulent discharge, significantly impacting patients’ quality of life. The disease is often associated with mental health challenges such as depression, anxiety, stigmatization, workplace difficulties, and even suicidal ideation [14].

The etiopathogenesis of HS is complex and poorly understood. It involves intricate interactions between genetic predispositions, environmental factors, and immune system dysregulation [15]. Obesity is a significant predisposing and exacerbating factor in HS, contributing to the frequent co-occurrence of metabolic syndrome and type II diabetes in affected individuals [16].

**Figure 2:**
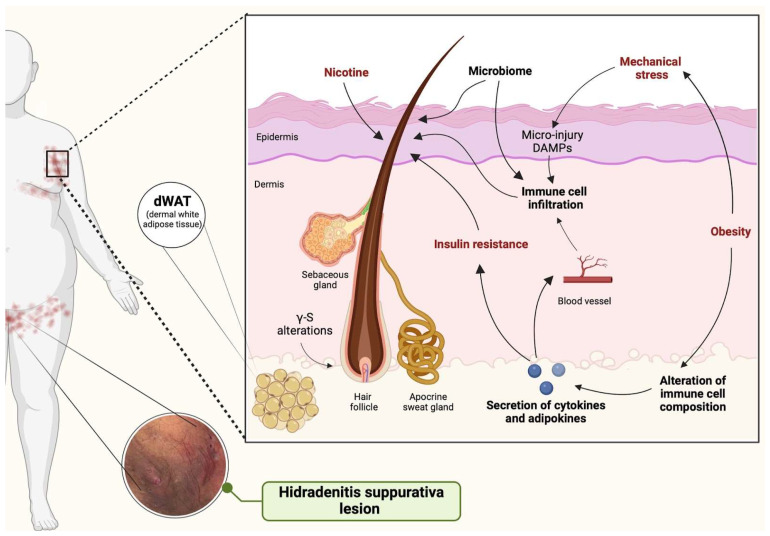
Graphical presentation of HS pathogenesis.

The HS inflammatory process begins with hyperkeratosis of terminal follicular orifices and hyperplasia of the follicular epithelium, leading to the obstruction of follicular and sebaceous units and subsequent immune system activation [13]. This triggers an inflammatory response within the skin, driven predominantly by pro-inflammatory cytokines such as IL-1βand TNF-α, along with mediators from activated Th1 and Th17 helper cells (including IFN-γ and IL-17), and effector mechanisms involving neutrophils, macrophages, and plasma cells [17,18,19]. A graphical presentation of HS pathogenesis is demonstrated in Figure 2.

### 3.1. Hidradenitis Suppurativa and Obesity

There is a well-established bidirectional relationship between obesity and HS, primarily driven by chronic low-grade inflammation and immune system dysregulation [20]. Studies have shown that skin samples from HS patients with a BMI greater than 30 exhibit elevated levels of molecular markers associated with metabolic disorders and obesity, including irisin protein, PPARγ, and IGF-1R [21,22]. Additionally, these patients often display increased expression of the IL-17 receptor, a key pro-inflammatory cytokine involved in HS pathogenesis and a marker of systemic inflammation [21].

High BMI exacerbates the severity and extent of skin lesions in HS patients [23]. The increased adipose tissue associated with obesity leads to greater friction between skin folds, enhancing the occlusion of follicular and sebaceous units and further promoting the expression of pro-inflammatory cytokines [24,25]. Moreover, obesity alters the molecular structure of collagen, disrupting skin integrity and leading to reduced mechanical strength and impaired wound healing [26]. This compromised skin barrier not only aggravates existing lesions but also impedes their remission, making significant obesity a major limiting factor in disease management [27]. Multiple studies have shown that the prevalence of HS is significantly higher in obese individuals, with estimates indicating that nearly 18–20% of obese patients may suffer from this condition, a rate much higher than the general population’s 1–2% prevalence [28,29].

Weight loss, particularly through bariatric surgery, has been demonstrated to substantially reduce the severity and prevalence of HS symptoms. A study by Kromann et al. [28] showed that a significant reduction in body weight (more than 15%) led to a 35% decrease in the number of patients reporting HS symptoms, with the average number of involved sites dropping from 1.93 to 1.22. Another study by Canard et al. [30] highlighted that bariatric surgery, particularly sleeve and bypass surgeries, resulted in a 69% reduction in the number of anatomical sites affected by HS and a 50% decrease in HS activity, as measured by a visual analog scale (VAS). This improvement was significantly greater than in patients who only received nutritional care [30]. Moreover, these weight loss interventions not only reduced the physical burden of HS but also improved patients’ quality of life. The Dermatology Life Quality Index (DLQI) scores significantly decreased in patients who underwent bariatric surgery compared to those who did not, underscoring the importance of weight management as a critical component of HS treatment [28,30].

### 3.2. Hidradenitis Suppurativa and Diabetes Mellitus

A significant association between HS and diabetes mellitus (DM) has been demonstrated in various studies. In a comprehensive systematic review and meta-analysis conducted by Bui et al. [31] in 2017, which included 107,050 patients from 14 studies in the systematic review and 104,373 patients from 7 studies in the meta-analysis, it was observed that the prevalence of diabetes was markedly higher in patients with HS (10.6%) compared to those without HS (3.8%). The analysis further revealed that HS patients had a threefold increased risk of developing diabetes compared to the general population [31].

Additionally, a study by Rached et al. [32], involving 99 HS patients, identified several significant factors contributing to the development of diabetes in this population. Notably, patients at Hurley stage III had a 5.3-fold higher risk of developing type II diabetes compared to those at Hurley stages I and II. Other factors such as older age and higher BMI were also associated with an increased risk of diabetes in HS patients. These findings underscore the importance of early diabetes screening and management in patients with severe HS, particularly those with advanced disease stages and other risk factors [32].

### 3.3. Pro-Inflammatory Mechanisms of Obesity in HS

In obese individuals, increased adipose tissue volume exacerbates HS lesions by creating a pro-inflammatory environment [33]. Adipose tissue harbors immune cells that produce pro-inflammatory mediators, leading to an influx of neutrophils and macrophages. This elevated immune cell activity induces oxidative stress and systemic low-grade inflammation, which accelerates the progression of HS lesions [33,34,35]. Additionally, the conditions within the skin folds of overweight individuals—such as moisture, friction, maceration, and an anaerobic environment—further contribute to lesion development [36].

Studies have shown that patients with HS exhibit decreased levels of the anti-inflammatory adipokine adiponectin and increased levels of leptin, resistin, and visfatin compared to healthy individuals. These elevated adipokine levels not only promote the development of obesity but also correlate with metabolic dysregulation [37]. Multivariate regression analysis has demonstrated a positive correlation between HOMA-IR and resistin levels and a negative correlation with adiponectin levels, indicating a pathogenic immune–metabolic system influenced by adipokines [36].

In a 2023 study by Witte et al. [36], it was found that the expression of genes involved in oxidative phosphorylation was downregulated in CD4+ T cells in the blood of HS patients, suggesting a metabolic shift towards glycolysis in HS. Obese individuals also have elevated levels of free fatty acids (FFAs), which negatively impact carbohydrate and lipid metabolism, thereby increasing the risk of cardiovascular diseases [36]. Furthermore, studies have reported higher incidences of non-alcoholic fatty liver disease (NAFLD), dyslipidemia, hyperglycemia, and diabetes in HS patients compared to healthy controls [36,38].

FFAs can induce the production of pro-inflammatory cytokines such as IL-1β, IL-6, and IL-8 by binding to the TLR4 receptor on monocytes, thereby enhancing inflammation [36,38]. Activation of IL-1RI by FFAs further disrupts normal glycemia and insulin secretion. Additionally, elevated glucose levels contribute to increased expression of FFA-induced pro-inflammatory factors within pancreatic islets [36,39].

These metabolic abnormalities significantly contribute to the reduced life expectancy observed in HS patients. A study of the Finnish population found that HS patients had a life expectancy 14.7 years shorter than the control group, with cardiovascular disease being the leading cause of death. The development of diabetes in HS patients is particularly associated with advanced Hurley stage III, older age, and higher BMI, with patients at Hurley stage III having a markedly increased risk of developing type II diabetes compared to those at Hurley stages I and II [40].

## 4. Glucagon-like Peptide-1 (GLP-1) Mechanism of Action

GLP-1 is an endogenous incretin hormone that plays a pivotal role in glycemic control and exerts pleiotropic effects across various metabolic pathways [41]. Primarily secreted by the L cells of the small intestine and colon in response to nutrient ingestion, particularly glucose, GLP-1 binds to its specific receptors (GLP-1R), which are part of the G-protein-coupled receptor family [42,43]. This binding initiates a cascade that activates adenylate cyclase, leading to an increase in intracellular cyclic adenosine monophosphate (cAMP) in pancreatic β-cells, thereby enhancing insulin secretion in a glucose-dependent manner [42].

GLP-1 enhances insulin secretion and contributes to pancreatic health by promoting the proliferation of β-cells and inhibiting their apoptosis [41]. This effect is critical for maintaining pancreatic function and mass, particularly in conditions of metabolic stress. Moreover, GLP-1 has a direct impact on pancreatic β-cells within the islets of Langerhans, stimulating insulin secretion and reducing glucagon release, which collectively helps to maintain glucose homeostasis and protect against postprandial hyperglycemia [44,45].

In addition to its effects on pancreatic cells, GLP-1 also slows gastrointestinal motility, leading to delayed gastric emptying, which contributes to a prolonged feeling of satiety [42]. This effect on gastric emptying, coupled with its action on GLP-1R in the hypothalamus—an area of the brain that regulates hunger and satiety—results in reduced food intake, making GLP-1 an important factor in weight management and obesity treatment [44,45].

Furthermore, GLP-1 has cardioprotective, neurotropic, and anti-inflammatory properties [46,47,48,49]. It enhances glucose uptake in skeletal muscle, thereby improving overall metabolic health [48]. Dysregulation of GLP-1 secretion, whether reduced or excessively high, can lead to metabolic disturbances, including obesity and reactive postprandial hypoglycemia. Research indicates that GLP-1 also has significant effects on cardiovascular function, including the modulation of heart rate and blood pressure, which further underscores its therapeutic potential beyond glycemic control [41].

## 5. GLP-1 Analogs

Endogenous and exogenous GLP-1 are characterized by a short half-life due to rapid inactivation by dipeptidyl peptidase-4 (DPP-4), an enzyme widely expressed in various cells and commonly found in the bloodstream [50]. This metabolic instability and brief circulation time limit GLP-1’s effectiveness as a therapeutic agent [51]. GLP-1 analogs, synthetically produced recombinant polypeptides, mimic the activity of GLP-1 by exhibiting agonistic effects [52]. These analogs, derived from exendin or human GLP-1, are engineered for enhanced resistance to proteolytic degradation by DPP-4 and possess slower elimination kinetics, thereby extending their duration of action [1,52].

The primary mechanism of GLP-1 analogs involves increasing insulin synthesis in response to hyperglycemia, reducing glucagon secretion in both hyperglycemic and euglycemic states, and slowing gastric emptying [1]. Additionally, these substances contribute to weight loss by acting on appetite centers in the hypothalamus [53]. For instance, Liraglutide, a long-acting GLP-1 receptor agonist (GLP-1 RA), facilitates weight reduction by stimulating the glutamatergic neural network, which contains the GLP-1 receptor [50].

**Figure 3:**
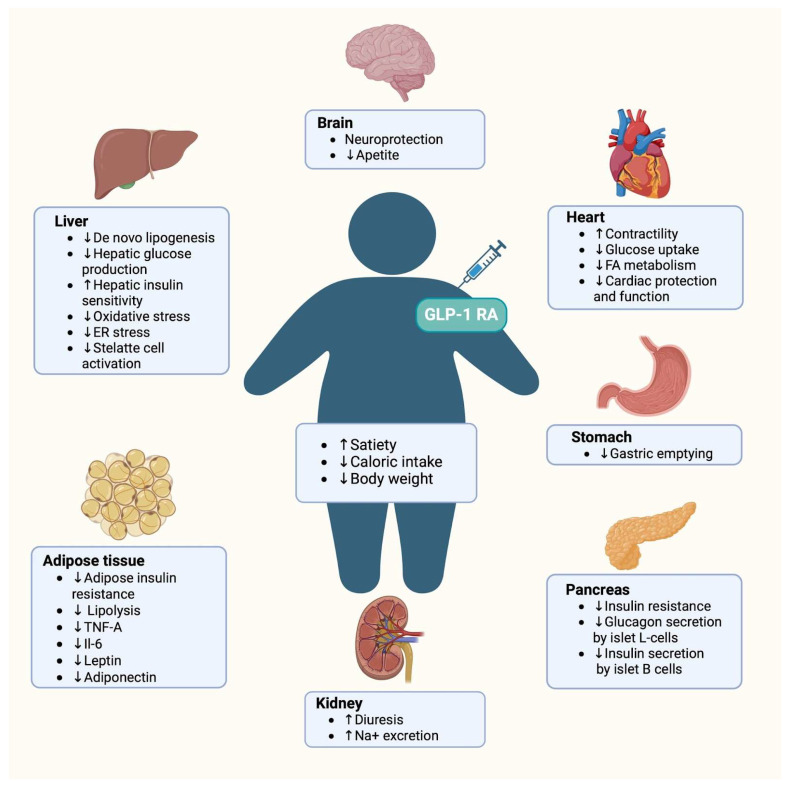
Graphical presentation of the mechanism of action of GLP-1 receptor analogs.

Beyond metabolic benefits, GLP-1 RAs exhibit cardioprotective effects, reducing the risk of cardiovascular events such as stroke and myocardial infarction [53]. They also display direct vasodilatory effects, which have been observed in individuals with obesity and type II diabetes [51]. The mechanism of action of GLP-1 RAs is presented in Figure 3.

### 5.1. Immunological Effects of GLP-1 RAs

GLP-1Rs are expressed in various subpopulations of immune cells, allowing GLP-1 RAs to exert significant immunomodulatory effects [54]. These agents exhibit anti-inflammatory properties primarily by inhibiting tumor necrosis factor-alpha (TNF-α), a key mediator in the synthesis of pro-inflammatory cytokines [55]. TNF-α plays a crucial role in the pathogenesis of inflammatory diseases, including HS, as it promotes the expression of nuclear factor kappa B (NF-κB), thereby exacerbating inflammation [54,55].

Invariant natural killer T (iNKT) cells, although constituting less than 1% of all peripheral blood T cells, play a pivotal role in the pathogenesis of inflammatory diseases [56]. The activation of iNKT cells increases the expression of pro-inflammatory cytokines such as IFN-γ, TNF-α, and IL-17, which can intensify the severity of skin lesions [56,57]. Notably, studies have shown that GLP-1 RA therapy can reverse the iNKT cell ratio in psoriatic lesions and circulation, leading to a reduction in the number of these cells within plaques and an increase in their presence in peripheral blood [56]. GLP-1 RA receptor activation also influences the proliferation and migration of T lymphocytes, thereby reducing inflammation [54].

Research has demonstrated that GLP-1 RAs, through their agonistic activity, inhibit chemokine-induced migration of human CD4+ lymphocytes in vitro by blocking PI-3 kinase activity [58]. A study by A. Faurschou et al. [59] revealed that GLP-1R expression is evident in the skin during inflammatory diseases characterized by immune cell infiltration. This finding is underscored by the absence of GLP-1R in human keratinocyte cultures [59]. Furthermore, GLP-1 RA agonists have shown beneficial effects on wound healing by reducing matrix metalloproteinase-9 (MMP-9) levels and the MMP-9/TIMP ratio within skin lesions, markers associated with earlier wound healing. They also contribute to the reduction in elevated C-reactive protein (CRP) levels, a common feature in inflammatory diseases [60].

### 5.2. The Use of GLP-1 RAs in HS

Liraglutide is a long-acting GLP-1 analog primarily used in the management of diabetes and non-diabetic overweight or obesity [61]. It enhances glucose-dependent insulin secretion, reduces plasma glucagon levels, delays gastric emptying, and modulates the hypothalamic hunger centers, leading to appetite suppression [62]. With a biological half-life of 13 h, liraglutide can be administered once daily to manage glycemia without the risk of hypoglycemia, as it does not cause glucose levels to drop below normal [3,63].

In a prospective case series by Nicolau et al. [64], 14 patients with both HS and obesity were treated with liraglutide at a dose of 3 mg for 3 months. The study reported significant reductions in BMI (from 39.3 ± 6.2 to 35.6 ± 5.8; *p* = 0.002), waist circumference (from 121.3 ± 19.2 cm to 110.6 ± 18.1 cm; *p* = 0.01), and systemic inflammatory markers, including C-reactive protein (CRP) (from 4.5 ± 2.2 mg/L to 3.0 ± 2.1 mg/L; *p* = 0.04), homocysteine (from 16.2 ± 2.9 µmol/L to 13.3 ± 3.0 µmol/L; *p* = 0.005), and plasma cortisol (from 15.9 ± 4.8 µg/dL to 12.6 ± 4.5 µg/dL; *p* = 0.007). The severity of HS lesions was assessed using the Hurley Staging System, which showed a marked improvement from a mean score of 2.6 ± 0.5 to 1.1 ± 0.3 (*p* = 0.002). Additionally, quality of life, measured by the Dermatology Life Quality Index (DLQI), improved significantly (from 12.3 ± 2.8 to 9.7 ± 6.9; *p* = 0.04) [64].

Beyond weight reduction, liraglutide appears to play a crucial role in mitigating the inflammatory mechanisms underlying HS. It is thought to suppress the molecular expression of TNF-α and other pro-inflammatory cytokines and inhibit the activation of the nuclear factor κB (NF-κB) pathway [65,66]. Liraglutide also reduces the secretion of monocyte chemotactic protein 1, which in turn affects the migration and activation of monocytes and macrophages [65,67]. Furthermore, its use has been linked to the suppression of interleukins such as IL-17, IL-22, and IL-23, which are typically elevated in HS lesions [68,69]. The dual impact of liraglutide on both immune modulation and weight control suggests its potential efficacy in the therapeutic management of HS [3]. Its favorable safety profile, characterized by rare side effects, further supports its use as a treatment option [70].

Lyons et al. [71] recently published a study on the use of liraglutide in HS patients, underlining its usefulness in controlling HS-related inflammation. The study assessed the impact of semaglutide on disease control and quality of life in 30 patients with both obesity and HS. The methodology involved a retrospective analysis of data collected from June 2020 to March 2023, including BMI, weight, DLQI, frequency of flares, C-reactive protein (CRP), glucose, and HbA1c levels. The mean duration of semaglutide treatment was 8.2 months, with an average weekly dose of 0.8 mg. In addition to semaglutide, many patients were receiving other HS treatments, including adalimumab, infliximab, brodalumab, metformin, and spironolactone, reflecting the complex, multimodal management typical of HS [71]. Results showed that patients experienced significant weight reduction, with the mean body weight decreasing from 117.7 kg to 111.6 kg (95% CI 2.88–9.29; *p* < 0.001). One-third of the patients lost at least 10 kg. BMI also decreased slightly, though the change was not statistically significant (from 43.1 to 41.5 kg/m^2^; *p* = 0.48). There was a notable improvement in quality of life, as the mean DLQI score improved from 13 to 9 (95% CI 1.70–10.68; *p* = 0.001), with one-third of patients achieving a clinically significant reduction of 4 points or more. The frequency of HS flares decreased from once every 8.5 weeks to once every 12.0 weeks, although this reduction did not reach statistical significance. Metabolic markers showed modest improvements, with HbA1c dropping from 39.3 to 36.6 mmol/mol (*p* = 0.03), but CRP levels did not significantly change [71]. The study concluded that semaglutide, even at relatively low doses, may help control HS by reducing weight and improving patients’ quality of life, though its effect on inflammatory markers and flare frequency was less pronounced. The rationale behind using semaglutide in HS stems from its ability to reduce body weight and modulate metabolic and inflammatory pathways, which are strongly linked to the severity and progression of HS. These results suggest semaglutide could be a useful adjunctive therapy in managing HS, but further placebo-controlled trials are necessary to confirm its efficacy.

**Figure 4:**
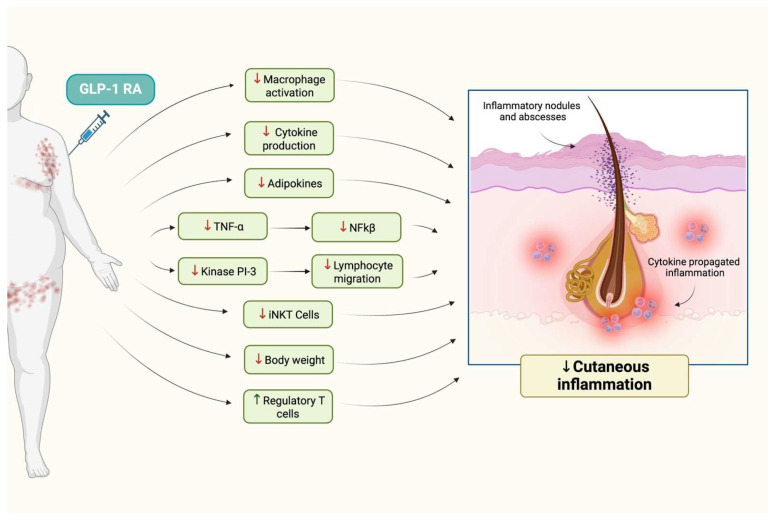
Graphical presentation of possible effects of GLP-1 RAs in HS.

While liraglutide and semaglutide are the only GLP-1 analogs currently used in HS treatment, other GLP-1 agonists, such as, dulaglutide, exenatide, and albiglutide, may also hold promise for future research and therapeutic use in HS. These medications share similar mechanisms of action and could offer alternative options pending further studies. Potential roles of GLP-1 RAs in HS are presented in Figure 4.

The use of GLP1-RAs in HS presents a promising therapeutic option, particularly before initiating biologics. GLP1-RAs, due to their anti-inflammatory and weight-reducing properties, could serve as an early intervention in HS, targeting both metabolic dysfunction and inflammation, which are critical in HS pathogenesis. This approach may help delay or reduce the need for biologic therapies, which are often expensive and associated with significant side effects (e.g., inflammatory bowel disease with IL-17 inhibitors). Additionally, the simultaneous use of GLP1-RAs and biologics could provide synergistic benefits by targeting different aspects of HS; GLP1-RAs address metabolic factors and systemic inflammation, while biologics specifically inhibit key inflammatory pathways like TNF-α or IL-17. This combination therapy may optimize disease management, particularly in patients with severe, refractory HS or those with significant metabolic comorbidities. However, further studies are necessary to fully establish the efficacy and safety of this combined approach.

## 6. Conclusions

In conclusion, GLP-1 receptor agonists, particularly liraglutide, demonstrate promising effects on HS. Liraglutide has shown the ability to reduce the severity of HS lesions by modulating key inflammatory pathways and contributing to weight reduction, which are critical in managing HS. The improvements in lesion severity and patient quality of life highlight the potential of GLP-1 agonists as effective treatments for this chronic condition.

While liraglutide and semaglutide are currently the only GLP-1 analogs used in HS, the potential for other GLP-1 agonists like dulaglutide to offer similar benefits warrants further investigation. Continued research will be essential to fully establish the role of GLP-1 agonists in the therapeutic management of HS.

## 7. Other Considerations

### 7.1. Psoriasis as a Disease Showing Similarities to HS

Like hidradenitis suppurativa, psoriasis is a chronic, recurrent inflammatory dermatosis [72]. Immune mechanisms are involved in its etiopathogenesis, leading to keratinocyte proliferation and functional stimulation. T lymphocytes, which release pro-inflammatory cytokines, play a particular role in this phenomenon [73]. Chronic inflammation in psoriasis leads to the frequent co-occurrence of obesity and other metabolic disorders such as diabetes and dyslipidemia [56,74].

GLP-1 RAs find use in the treatment of psoriasis due to their immunological effects. They reduce the secretion of pro-inflammatory cytokines induced by TNF-α, involved in the etiopathogenesis of the disease. TNF-α inhibition prevents the activation of the NF-κB signaling pathway associated with inflammation. In addition, GLP1 RAs block lymphocyte proliferation and CD4+ lymphocyte migration [55,75].

As in HS, a bidirectional relationship is observed between obesity and psoriasis. GLP-1 RA-induced weight reduction contributes to the suppression of inflammation caused by dysfunctional adipose tissue and enables improvement in the severity of psoriasis, particularly the severe forms [20].

In a meta-analysis involving 2,829,312 patients, the global prevalence of psoriasis and obesity comorbidity was 25%. In addition, a correlation was found between the prevalence of obesity and the severity of psoriasis (mild psoriasis—27%; moderate psoriasis—36%; severe psoriasis—95%) [76].

### 7.2. The Use of GLP-1 RAs in Psoriasis

Liraglutide significantly improves skin lesions in patients with psoriasis and excessive body weight. Its therapeutic potential is related to its anti-inflammatory, hypoglycemic, and weight-reduction effects [77]. A study involving 20 patients demonstrated a beneficial effect of GLP-1 RAs on the severity of psoriasis in obese patients [77]. Liraglutide at a dose of 3mg taken for three months enabled a reduction in BMI (38.9 ± 5.8 kg/m^2^ vs. 36.4 ± 5.6 kg/m^2^) and pro-inflammatory factors (CRP, homocysteine, ferritin, plasma cortisol) and improvements in DLQI (12.7 ± 7 points vs. 6.4 ± 5.6 points) and PASI (10 ± 8.4 points vs. 5.1 ± 6 points) [78]. In another study, seven patients with type II diabetes and psoriasis achieved a decrease in BMI from 23 ± 4 kg/m^2^ to 21 ± 3 kg/m^2^ after 12 weeks of treatment with liraglutide [77]. There were also improvements in DLQI (21.8 ± 6 points to 4.1 ± 3.9 points) and PASI (15.7 ± 11.8 points to 2.2 ± 3.0 points) [76]. In a randomized trial involving 25 patients with type II diabetes and psoriasis, a reduction in DLQI (22 ± 5.85 points to 3.82 ± 3.60 points) and PASI (15.7 ± 11 points to 2.2 ± 3 points) was observed after 12 weeks of treatment with liraglutide. In addition, inhibition of the expression of pro-inflammatory factors such as IL-17, IL-23, and TNF-α in psoriatic lesions was noted [79].

Semaglutide effectively lowers glucose levels and enables weight reduction. It is becoming a potential therapeutic agent through its additional anti-inflammatory effect, which is used in the treatment of patients with type II diabetes and psoriasis [80]. In a case report of a 73-year-old patient with type II diabetes, obesity, and plaque psoriasis, semaglutide enabled a 16.3% decrease in BMI [80]. Improvements in DLQI (26 to 0 points) and PASI (33.2 to 32 points) were also observed [81]. In another case report, a 50-year-old female patient with type II diabetes, abdominal obesity, and psoriasis received a 10-month treatment with semaglutide, which led to a significant reduction in body weight. There was an improvement in the patient’s quality of life (DLQI) and psoriasis lesion severity (PASI) [82].

Data on the treatment of psoriasis with exenatide are limited. However, M. Buysschaert et al. [83] described the case of a 61-year-old patient with type 2 diabetes and psoriasis who was started on exenatide therapy. Weight loss and improved glycemia were observed within a short time. Taking exenatide enabled a reduction in the severity of psoriatic lesions, which was reported as a decrease in PASI from 11 to 3–4 points [83].

## Data Availability

Not applicable.

## Abbreviations

GLP-1	Glucagon-Like Peptide-1
GLP-1R	Glucagon-Like Peptide-1 Receptor
GLP-1RA	Glucagon-Like Peptide-1 Receptor Agonist
HS	Hidradenitis Suppurativa
BMI	Body Mass Index
DLQI	Dermatology Life Quality Index
CRP	C-Reactive Protein
IL-1β	Interleukin-1 Beta
TNF-α	Tumor Necrosis Factor-Alpha
IL-17	Interleukin-17
NF-κB	Nuclear Factor Kappa B
MMP-9	Matrix Metalloproteinase-9
PASI	Psoriasis Area Severity Index
DPP-4	Dipeptidyl Peptidase-4
VAS	Visual Analog Scale
IFN-γ	Interferon Gamma
Th1/Th17	T Helper Cells 1/17
NAFLD	Non-Alcoholic Fatty Liver Disease
FFAs	Free Fatty Acids
HOMA-IR	Homeostatic Model Assessment for Insulin Resistance
MCP-1	Monocyte Chemoattractant Protein-1
